# Reintegration Into the Workforce After Kidney Transplantation Based on Urbanization Status in Switzerland

**DOI:** 10.1016/j.ekir.2024.10.029

**Published:** 2024-11-06

**Authors:** Federica Bocchi, Selina Müller, Isabelle Binet, Dela Golshayan, Fadi Haidar, Thomas Müller, Stefan Schaub, Aurelia Schnyder, Daniel Sidler, Federico Storni, The Swiss Transplant Cohort Study, Patrizia Amico, Adrian Bachofner, Vanessa Banz, Sonja Beckmann, Guido Beldi, Christoph Berger, Ekaterine Berishvili, Annalisa Berzigotti, Pierre-Yves Bochud, Sanda Branca, Heiner Bucher, Anne Cairoli, Emmanuelle Catana, Yves Chalandon, Sabina De Geest, Sophie De Seigneux, Michael Dickenmann, Joëlle Lynn Dreifuss, Michel Duchosal, Thomas Fehr, Sylvie Ferrari-Lacraz, Jaromil Frossard, Christian Garzoni, Déla Golshayan, Nicolas Goossens, Fadi Haidar, Jörg Halter, Dominik Heim, Christoph Hess, Sven Hillinger, Hans Hirsch, Patricia Hirt, Linard Hoessly, Günther Hofbauer, Uyen Huynh-Do, Franz Immer, Michael Koller, Andreas Kremer, Thorsten Krueger, Christian Kuhn, Bettina Laesser, Frédéric Lamoth, Roger Lehmann, Alexander Leichtle, Oriol Manuel, Hans-Peter Marti, Michele Martinelli, Valérie McLin, Katell Mellac, Aurélia Merçay, Karin Mettler, Nicolas Müller, Ulrike Müller-Arndt, Beat Müllhaupt, Mirjam Nägeli, Graziano Oldani, Manuel Pascual, Jakob Passweg, Rosemarie Pazeller, Klara Posfay-Barbe, David Reineke, Juliane Rick, Anne Rosselet, Simona Rossi, Rössler, Silvia Rothlin, Frank Ruschitzka, Thomas Schachtner, Stefan Schaub, Alexandra Scherrer, Dominik Schneidawind, Aurelia Schnyder, Macé Schuurmans, Simon Schwab, Thierry Sengstag, Federico Simonetta, Jürg Steiger, Guido Stirniman, Ueli Stürzinger, Christian Van Delden, Jean-Pierre Venetz, Jean Villard, Julien Vionnet, Madeleine Wick, Markus Wilhlem, Patrick Yerly

**Affiliations:** 1 Department of Nephrology and Hypertension, Inselspital, Bern University Hospital, Bern, Switzerland; 2 Department of Visceral Surgery and Medicine, Inselspital, University Hospital Bern, Bern, Switzerland; 3 Departement of Nephrology and Transplantation Medicine, Kantonspital St. Gallen, St. Gallen, Switzerland; 4 Transplantation Center, Department of Medicine, Lausanne University Hospital (CHUV), Lausanne, Switzerland; 5 Department of Medicine, Division of Nephrology, University Hospital of Geneva (HUG), Geneva, Switzerland; 6 Department of Nephrology, USZ, University Hospital Zurich, Zurich, Switzerland; 7 Departement of Transplantation Immunology and Nephrology, University Hospital Basel, Basel, Switzerland

**Keywords:** graft outcomes, kidney transplantation, urbanization, work capacity, work reintegration

## Abstract

**Introduction:**

Most of Switzerland’s population and employment opportunities are concentrated in urban areas. Although kidney transplantation (KT) is the preferred therapy for eligible candidates, individuals in rural areas face challenges accessing specialized medical services due to longer travel distances. Limited understanding exists regarding patients' perspectives on returning to work after KT and whether this correlates with their urbanization status, potentially influencing outcomes.

**Methods:**

Retrospective, nationwide (Swiss Transplant Cohort Study [STCS]) study, from May 2008 to 2020, including 1926 patients aged 18 to 60 years who underwent KT. We investigated the self-reported work reintegration at 1, 3, and 5 years after the KT, the recipient and allograft survival, and the allograft function at 12 months, contingent on urbanization status (urban, suburban, rural).

**Results:**

The return rate of sufficiently filled-in questionnaires was 81% (1053 participants). Urban recipients were younger, had longer dialysis time before KT, and had less living donor KT. At baseline, the level of education, as well as the workforce defined as work capacity > 50%, were lower in urban areas (37% urban, 39% suburban, and 47% rural). Regression analysis revealed significantly higher odds ratio for employment 1 year post-KT among patients residing in rural and suburban areas (odds ratio: 1.31 [confidence interval: 1.04–1.65] and 1.52 [confidence interval: 1.16–1.98], respectively) compared to patients from urban regions. Stratified according to urbanization environment, recipient and allograft survival were comparable across groups.

**Conclusion:**

Patient and graft outcomes were favorable, with improved work reintegration observed at the 1-year mark post-KT for recipients from rural backgrounds compared to those from suburban and urban areas.

## Graphical abstract

**Figure:**
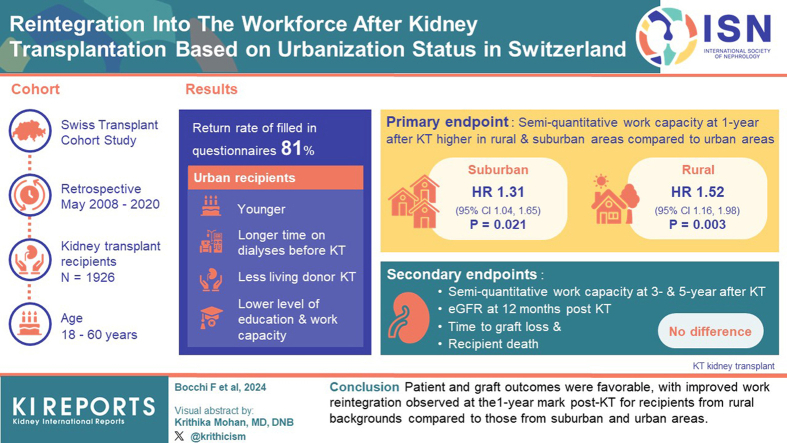

The Alps cover 60% of Switzerland's land area, but only 11% of the population (approximately 8.9 million) lives there, with nearly 75% of Swiss residents concentrated in urban areas where 80% of employment is found.^1^ Individuals residing in peripheral, rural, or mountainous areas often face longer distances to access hospitals offering specialized care.^2^ Despite Switzerland's health care system being structured to ensure equal access, disparities in infrastructure, pose challenges for these populations.^3^

Compared to hemodialysis (HD), KT is the favored renal replacement therapy for eligible individuals with end-stage renal disease, leading to enhanced survival, decreased morbidity, and an improved quality of life.^4^ Graft survival rates exceed 90% at 1 year, over 70% at 5 years, and 60% at 10 years.^5^^,^^6^ Health-related quality of life and ability to work are key measures of treatment success. In Switzerland, KT is performed in 6 transplant centers, whereas approximately 4500 individuals undergo HD at 100 centers, with 90% receiving in-center HD.^7^ Over the past 15 years, approximately 3800 KTs have been performed, two-thirds from deceased donors.^8^

Longer travel distance and limited public infrastructure in rural regions may hinder access to KT and could influence patient and allograft outcome.^9^ Spatial information has been used to assess patient outcome in nephrology, surgery, and transplantation, with studies linking distance to transplant centers with access to waitlist placement and outcomes in the US and UK.^10^^,^^11^

The ability to return to work after a KT is influenced by various sociodemographic, clinical, health-related, and work-related factors.^6^ Maintaining full-time employment from the time of transplantation through the first year is linked to lower risks of graft failure and recipient mortality.^6^^,^^12^ Conversely, research suggests that remoteness from medical facilities can impede socioeconomic reintegration following complex medical procedures.^13^^,^^14^

In this study, we employed data from a multicenter comprehensive KT nationwide prospective cohort to evaluate work capacity at 1 year after KT as primary end point, based on urbanization status. In addition, we examined the following: (i) self-reported work reintegration at 3 and 5 years post-KT, (ii) recipient and allograft survival, and (iii) allograft function at 12 months.

## Methods

### Study Design and Population

The STCS (www.stcs.ch) is a multicenter nationwide cohort study conducted in Switzerland. The STCS prospectively enrolls all solid organ transplantations at all 6 Swiss transplant centers since May 2008. This study is an analysis of registry data from May 5, 2008 to December 28, 2020 and includes all adult patients who received a KT at the university hospital of Basel, Bern, Geneva, Lausanne, St. Gallen, and Zürich, Switzerland.^8^ The study was approved by STCS scientific committee (FUP156) and the Medical Ethics Committee of the study centers (ID 2017 01267), and informed consent was obtained from all the participants at enrollment in the study.

### Covariates

Recipient characteristics were collected from the STCS database. Recipient age, sex, body mass index (in kg/m^2^), kidney and previous transplant history (cause of kidney disease, time spent on dialysis [HD or peritoneal dialysis, in years], preemptive KT, previous KT, and donor type [living or deceased donor KT]) were recorded. Kidney diseases were classified as vascular and/or diabetic nephropathy as being associated with a worse outcome (as opposed to other nephropathies leading to KT). Work-related information was gathered using questionnaires. We analyzed the work capacity in addition to the level of education, marital status, and household income at the time of KT (baseline). Work capacity was captured as self-reported work load between 0% and 100% (0%, 0%–20%, 21%–50%, 51%–80%, >81%). An unemployed person was defined as 0% employment, whereas >81% was equivalent to full-time work. For brevity, the following baseline covariates were dichotomized: education was categorized as lower (no degree, mandatory, vocational) and higher (higher professional education, high school, university); work capacity at baseline as low (0%–50%) and high (>50%); and marital status as married (married, in a relationship) and single (single, divorced, widowed). Coordinates of recipient’s domicile (at time of KT), and of Swiss HD and KT centers were documented. Recipient’s community’s degree of urbanization (urban, suburban, rural) was extracted for all Swiss communities from Eurostat and assigned to each recipient based on registered domicile coordinates.^15^^,^^16^ Travel distance in kilometers and travel time in minutes from domicile to the closest HD and KT center were calculated using the Google Maps Distance API tool. To calculate travel distance and travel time, the following parameters were chosen: mode: driving; departure time: 8 am; and traffic model: best guess.

### Primary and Secondary End Points

The primary end point of the study was the work capacity at 1 year after KT measured as described above (between 0% and 100% [0%, 0%–20%, 21%–50%, 51%–80%, >81%]). Secondary end points were the work capacity at 3 and 5 years after KT, estimated glomerular filtration rate (eGFR) at 12 months post-KT, time to graft loss or recipient death. eGFR, given in ml/min per 1.73 m^2^ was calculated using the Chronic Kidney Disease Epidemiology Collaboration 2009 creatinine equation.^17^ For patients with graft loss before 12 months, eGFR was set to at 0 ml/min per 1.73 m^2^.

### Exclusion Criteria

Participants were excluded from the analysis if they met any of the following criteria: (1i) aged <18 or >60 years at the time of KT; (ii) residing outside Switzerland; (iii) having incomplete baseline data, including lacking domicile information; (iv) receiving a multiorgan transplantation; and (v) refusing or withdrawing informed consent.

### Patient and Public Involvement

Neither patients nor the public were involved in the design, conduct, or analysis of this study.

### Statistical Analysis

Results were reported as number of participants (percentage) for categorical data and median (interquartile range [IQR]) for continuous data. For model selection, demographic and clinical parameters were chosen and divided into confounders and mediators, with only the confounders selected for later modelling (age, sex, higher education, marital status, and work capacity at baseline). Medical history (kidney disease, dialysis time, preemptive or previous KT and donor type) were not used because these factors are likely mediators on the causal pathway between urbanization and outcomes (1-year work capacity, composite of death and graft loss; Supplementary Figure S1A and B) and may introduce overadjustment bias. We used the following parameters for the generalized linear regression models: the response variable was work integration at 1, 3, and 5 years, respectively (0%, 0%–20%, 21%–50%, 51%–80%, >81%, treated as a ordinal variables); the prediction variables for the reduced models included urbanization (urban, suburban, rural). For the full models, the prediction variables were urbanization (urban, suburban, rural), age (per decade), sex (male), marital status (married), and work capacity at baseline (>50%). For the composite end point of graft loss and death, Kaplan Meier curves were drawn with log rank test. For the individual end points of graft loss or death, a competing risk analysis was conducted using cumulative incidence curves and Fine-Gray subdistribution analysis shown as Forrest plots. eGFR at 12 months was plotted with Jitter plots and analyzed with Mann Whitney U test. eGFR slope was calculated from eGFR trend over time for each participant after 12 months post-KT until death, graft loss or censoring (given in ml/min per 1.73 m^2^ per year). eGFR slopes were plotted for individual groups with boxplots and analyzed with Mann Whitney U test. A 2-tailed *P* < 0.05 was considered statistically significant. Statistical analyses were performed using R (version 4.0.3) and R Studio (version 1.3.1093) (R Foundation for Statistical Computing, Vienna, Austria) and the following packages: tidyverse, nephron, cowplot, patchwork, survival, and sf.

## Results

### Selection Procedure and Overall Characteristics of Participants

**Figure 1:**
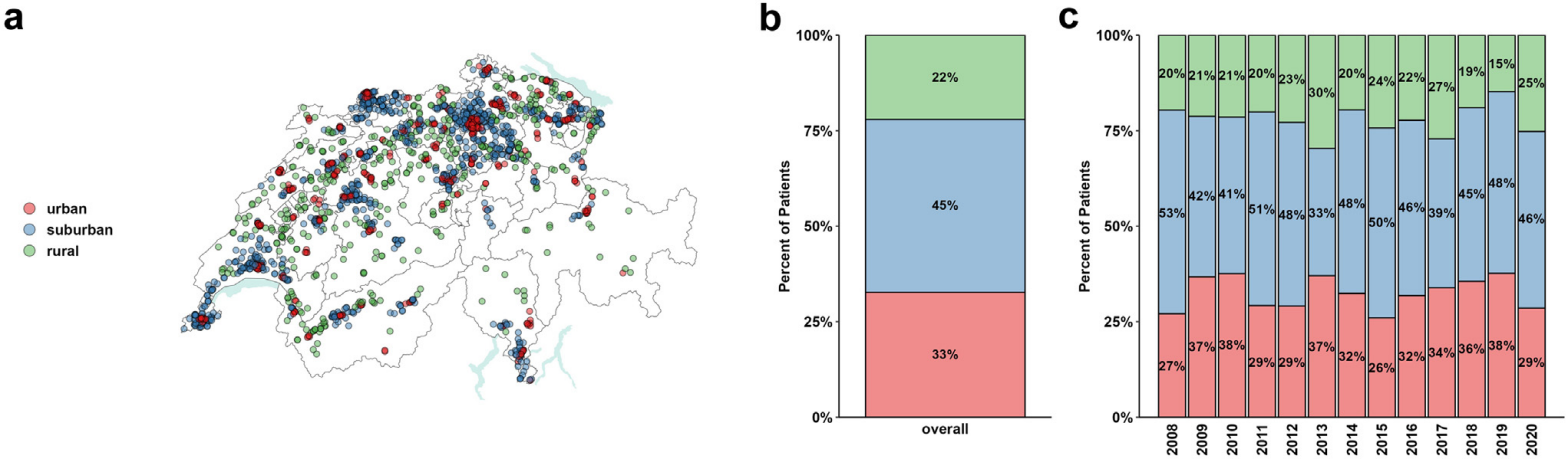
(a) Urbanization environment of KT recipients. The map shows Swiss cartography with cantonal areas. Recipient domicile is plotted. Color codes indicate urban (red), suburban (blue), and rural (green) environments. (b, c) Proportional frequency of patients from urban (red), suburban (blue), and rural environments (green) for the entire study period (B) and resolved for KT year (C). KT, kidney transplantation.

**Figure 2:**
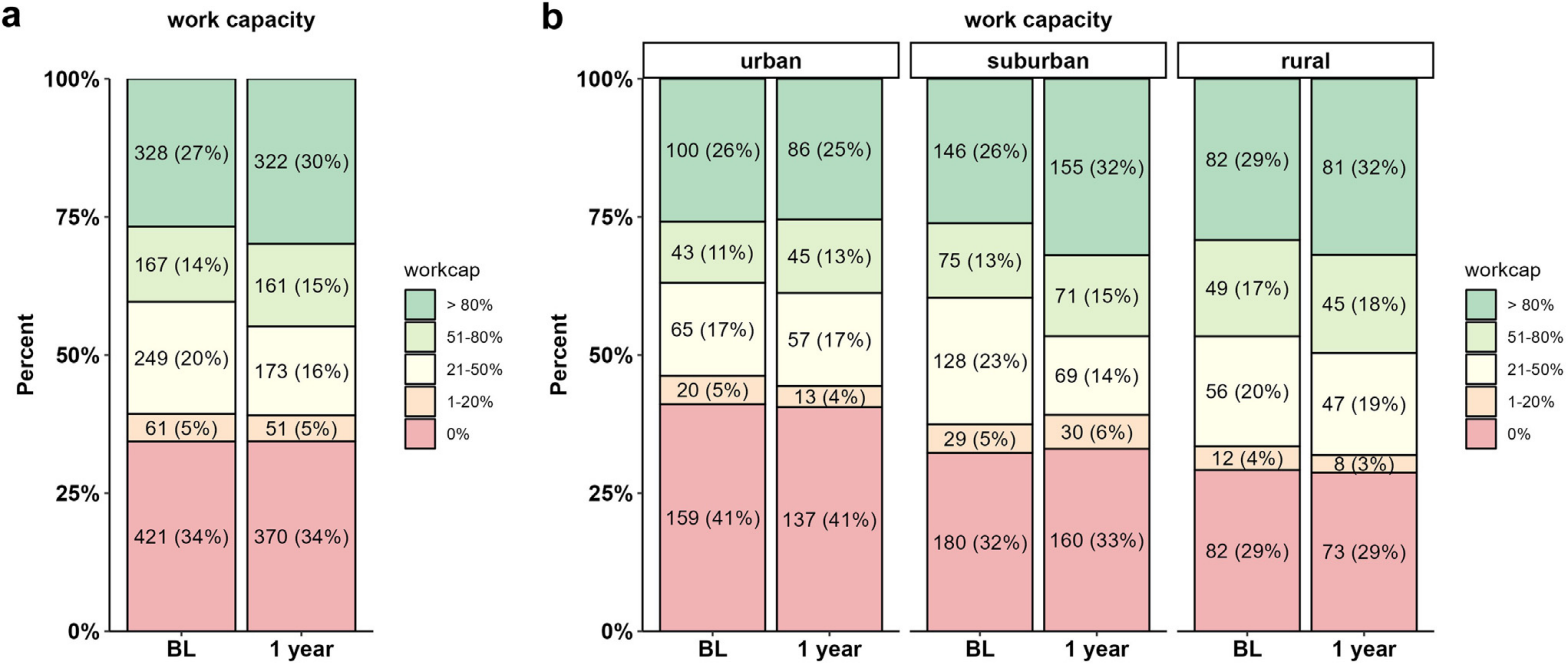
Work capacity at baseline and 1-year post-KT. (a) Overall results and (b) stratified according to urbanization status (urban, suburban, rural). BL, baseline; KT, kidney transplantation.

A total of 1926 KT recipients were included in the final analysis, following selection based on exclusion criteria (Supplementary Figure S2). The median follow-up time was 5.0 years (IQR: 2.1–8.0 years; maximum: 12.3 years). In Table 1, we provide an overview of the characteristics of the included participants. In Figures 1 and 2, we illustrate the distribution of KT participants based on the recipients' place of residence in Switzerland, categorized into urban, suburban, and rural areas; and their work capacity at baseline and 1 year post-KT, respectively. Of the participants, 630 (33%) resided in urban areas, 871 (45%) in suburban areas, and 425 (22%) in rural areas at the time of KT. Participants living in urban areas were younger than those from suburban or rural areas, had longer periods of dialysis before KT, and were less likely to receive living donor KT. In urban areas, the level of education was lower, work capacity at baseline and household income were lower, and patients were less likely to be married. Detailed information about marital status are presented in Supplementary Table S1.

**Table 1:** Baseline characteristics of KT recipients within the STCS between 2008 and 2020, stratified for recipient’s urbanization environment at baseline

Characteristics	Urbanization			
	Overall *N* = 1926	Urban *n* = 630 (33%)	Suburban *n* = 871 (45%)	Rural *n* = 425 (22%)
Age (yrs)	48 (38–55)	47 (36–54)	49 (40–55)	49 (38–55)
Sex (male)	1215 (63%)	395 (63%)	552 (63%)	268 (63%)
Medical history				
Kidney disease (vascular/diabetes)	583 (19%)	182 (19%)	281 (20%)	120 (18%)
BMI	24.7 (21.5–28.1)	24.3 (21.3–27.5)	25.0 (21.7–28.1)	24.8 (21.8–28.5)
Dialysis time (years)	2.4 (0.8–4.4)	2.7 (1.1–4.7)	2.3 (0.8–4.3)	1.9 (30.6–4.0)
Preemptive KT	373 (12%)	129 (13%)	173 (13%)	71 (11%)
Previous KT	330 (17%)	95 (15%)	148 (17%)	87 (20%)
Donor type (LDT)	862 (45%)	264 (42%)	381 (44%)	217 (51%)
Education and work load				
Higher education (yes)	513 (22%)	140 (19%)	247 (23%)	126 (23%)
Work capacity at BL (>50%)	499 (40%)	146 (37%)	221 (39%)	132 (46%)
Marital status				
Married / relationship	1268 (66%)	379 (60%)	580 (67%)	309 (73%)

BL, baseline; BMI, body mass index; KT, kidney transplantation; LDT, living donor transplantation; STCS, Swiss Transplant Cohort Study.

### Distance to Closest HD and KT Centers

**Figure 3:**
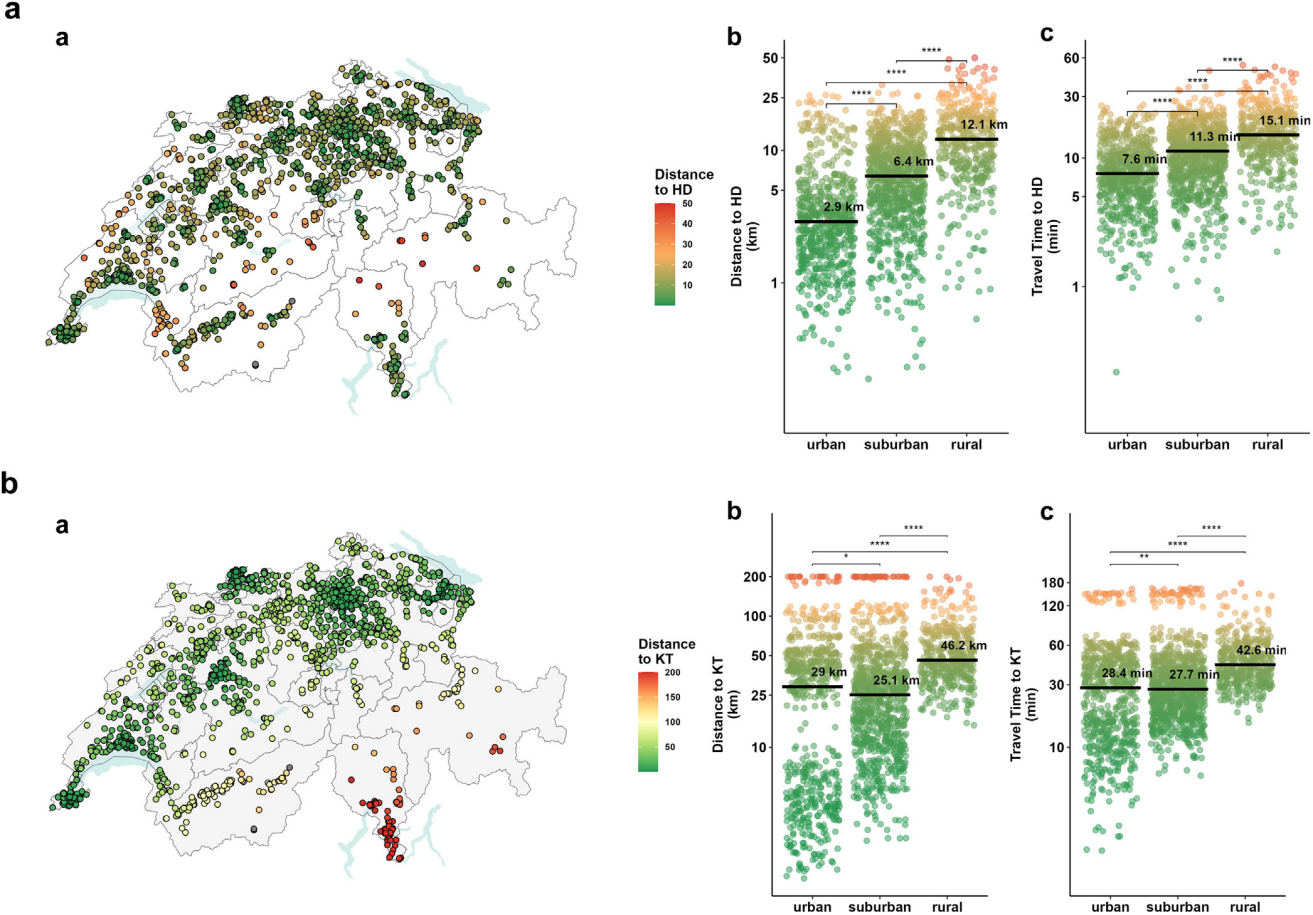
The map shows Swiss cartography with cantonal areas. Recipient domicile is plotted. Driving distance in kilometers and travel time in minutes to closest HD center (a, top) and to closest KT center (b, bottom). HD, hemodialysis; KT, kidney transplantation.

The median distance from home to the closest HD center was 5.7 (IQR: 2.8–10.9) km, reflecting a median of 10.7 (IQR: 6.8–15.1) minutes travel time. Between the groups, distance and travel time were notably greater in recipients residing in rural areas (12.1 km, 15.1 minutes) compared to those from suburban (6.4 km, 11.3 minutes) and urban areas (2.9 km, 7.6 minutes). Variance of distance and time was relatively small for all 3 groups, reflecting the dense and evenly distributed HD network in Switzerland (Figure 3a, Supplementary Table S2).

Regarding distance to the KT center, median travel distance was 32.5 (IQR: 11.6–52.1) km with a median travel time of 31.6 (IQR: 19.1–47.0) minutes. Rural recipients had the longest travel distance. Urban recipients, particularly in the south and southeastern regions where HD services are abundant, but KT centers are far, exhibited greater variability (Figure 3b, Supplementary Table S2).

### Work Capacity and Reintegration

The return rate of sufficiently filled-in questionnaires for work capacity, marital status, and household income was 68%, 79%, and 83% at 1, 3, and 5 years for patients who reached these milestones, respectively. Work capacity at 1 year post-KT was highest in rural and suburban areas compared to urban areas (Figure 2). The logistic regression analysis presented in Table 2, showed that recipients from rural and suburban residences exhibited significantly increased odds ratio (1.31 and 1.52, respectively) for higher employment when compared to recipients from urban areas in the minimal model. These results remained consistent even after accounting for other factors (full model). A sensitivity analysis of work integration at 3 and 5 years post-KT revealed similar findings, notably increased hazard ratio for work reintegration in suburban and rural areas. Significance was not universally reached because return rate of questionnaires were substantially lower for these time points (Supplementary Table S3A and B).

**Table 2:** Work load at 1 year after KT, assessed from a reduced model (urbanization) and a full model (urbanization, age, sex, higher education, marital status, income, and work capacity at baseline)

Analyzed parameters	Reduced model			Full model		
	OR	95% CI	*P*-value	OR	95% CI	*P*-value
Urbanization						
Urban	-	-		-	-	
Suburban	1.31	1.04–1.65	0.021	1.32	1.07–1.62	0.011
Rural	1.52	1.16–1.98	0.003	1.39	1.09–1.77	0.012
Recipient age (per decade)				0.74	0.68–0.81	<0.001
Recipient sex (male)				1.53	1.28–1.85	<0.001
Higher education (yes)				1.77	1.43–2.19	<0.001
Marital status (married)				1.21	1.00–1.46	0.054
Work capacity at BL (>50%)				4.83	4.00–5.83	<0.001

BL, baseline; CI, confidence interval; KT, kidney transplantation; LDT, living donor transplantation; OR, odds ratio.

### Recipient and Allograft Survival

**Figure 4:**
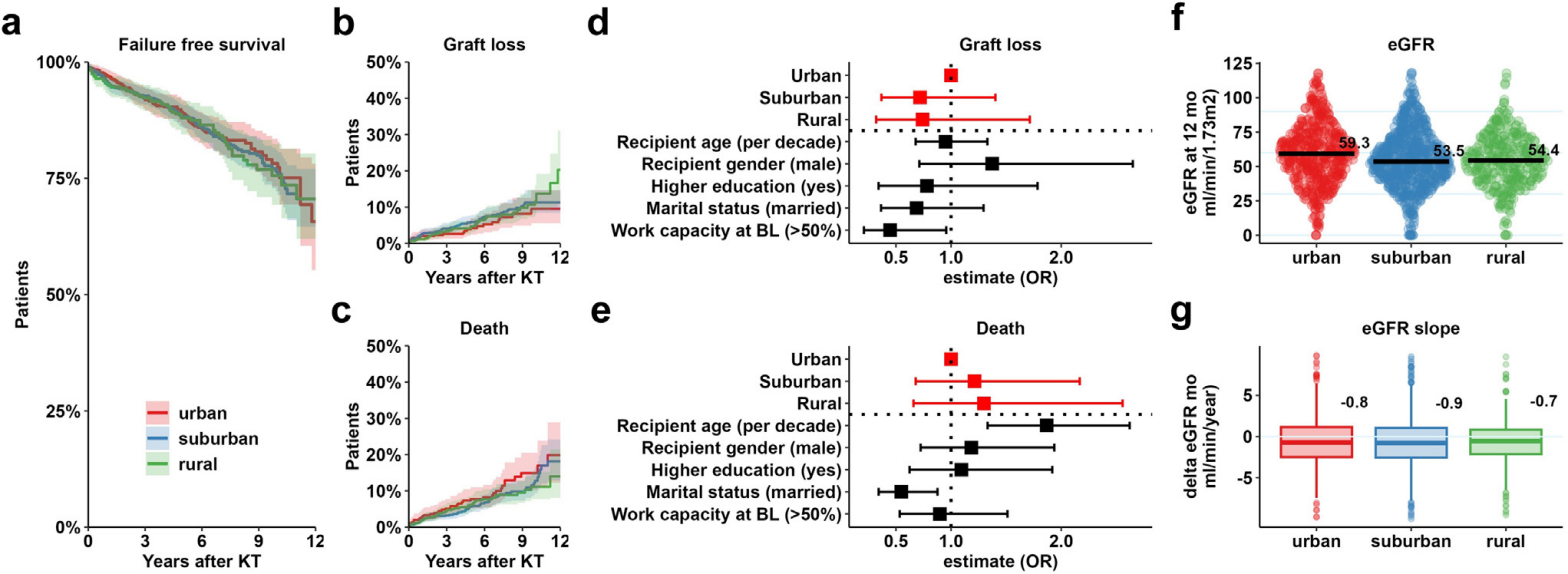
Recipient, allograft survival, and allograft function at 12 months. (a–c) Kaplan Meier curve for the composite end point of failure-free survival and cumulative incidence curves for graft loss and death. (d and e) Fine-grey subdistribution model for graft loss and death with correction for cofactors, including recipient age, sex, higher education, marital status, and baseline work capacity. (f and g) eGFR at 12 months and slope for urban, suburban, and rural participants. KT, kidney transplantation; OR, odds ratio.

Stratified according to urbanization environment, recipient and allograft survival were comparable across groups. Cumulative incidence curves demonstrate that events were equally distributed among the groups for the individual end points of graft loss or death (Figure 4a–c, Supplementary Table S4). In Fine-Gray sub-distribution model, recipient’s urbanization had no impact on the competing end points of graft loss or death after correction for cofactors, including recipient age, sex, higher education, marital status, monthly income and baseline work capacity (Figure 4d–e). The eGFR at 12 months was comparable among the groups, with values of 59.3, 53.5, and 54.4 ml/min per 1.73 m^2^ for urban, suburban, and rural recipients, respectively (Figure 4f). Finally, the decline in eGFR during the study was comparable across the 3 groups (Figure 4g).

## Discussion

In this study, we examined how work integration correlates with urban status (urban, suburban, and rural) among KT recipients. The ultimate goal was to assess self-reported work reintegration and determine recipient and allograft survival, along with allograft function at the 12-month mark. Regression analysis revealed significantly higher odds ratio for employment 1 year post-KT among patients residing in rural and suburban areas compared to patients from urban regions. Recipient and allograft survival were comparable across groups when stratified by urbanization environment.

The emphasis on employment retention or return to work has been increasingly highlighted in organ transplantation overall, largely due to the well-documented long-term survival rates.^6^^,^^18^ Indeed, patients' overall quality of life is significantly influenced by their ability to return to work and participate in social activities after transplantation.^18^^,^^19^ Studies conducted over the past few decades have explored the posttransplantation return to employment, revealing considerable variability. The findings vary significantly across different countries, with the highest percentage of post-KT return-to-work observed in Holland at 67%, followed by Italy, Finland, and the USA.^19^ This variation could be related to differing definitions of employment, as well as the diverse clinical characteristics and demographic factors of the populations studied.^20^ Based on a recently published study, employment rates in Switzerland seem to be even higher, hovering at about 70% at 1 year post-KT.^19^ Our present results are in high agreement and additionally provide specific spatial resolution. These percentages also differ based on the type of transplant, with the main predictor being the duration of time spent on the waiting list.^18^^,^^21^^,^^22^ Various others factors, including sociodemographic elements (younger age, higher educational attainment), clinical aspects (primary kidney disease, duration of HD, preemptive KT), health-related characteristics, and work-related factors, were also investigated. Results demonstrated that these factors influenced the maintenance of employment after KT.^20^^,^^23^^,^^24^

To our knowledge, very little is known about how urbanization affects the return to work after transplantation. In our study sample comprising 1926 KT recipients, of which 1053 (81%) completed work reintegration questionnaires, we observed that at 1 year post-KT, work capacity and integration were highest in rural and suburban areas compared to urban areas. Despite expectations, residing farther from the KT center does not appear to hinder the return to work. In fact, according to our results, rural areas tend to be home to people with more work skills. Our results remained unchanged even after analysis including other cofactors (age, sex, higher education, marital status, and baseline work capacity). This improved reintegration into the workplace could be explained by the fact that people in rural areas often have stronger social support networks, providing emotional and physical assistance during recovery, which aids in quicker recuperation. In addition, rural job opportunities tend to be less competitive, the environment less stressful and work schedules more accommodating, facilitating an easier return to work. Lastly, rural residents may exhibit greater resilience and adaptability due to the self-sufficiency and resourcefulness demanded by their environment.^24^, ^25^, ^26^

In addition, we demonstrate commendable and consistent clinical outcomes across recipients regardless of their urbanization status or proximity to KT centers. The eGFR at 12 months and this decline was comparable among the groups. For this reason, though there might be some hesitance, geographical location should not play a role in the decision-making process regarding KT. This is especially significant for our country, where distances are easily manageable, and a high density of medical centers is available, even in rural areas. To our knowledge, this study represents a pioneering effort in examining the process of reintegrating into work after KT within a highly detailed geographic context, while meticulously considering essential patient covariables.

Our study has several limitations. First, urbanization status was captured at time of KT and therefore does not account for patients who have moved during follow-up. Although our study comprehensively evaluated various parameters and involved all Swiss centers, it was not multinational, which limits the generalizability of our findings. Furthermore, Switzerland's small size and efficient transportation infrastructure make it relatively easy to overcome geographical distances, including job commuting. We believe that the outcomes could differ if the study was conducted in a larger country, or in large urban areas such as European or US metropoles. Are Switzerland's rural regions rural enough when compared with other countries on a broader geographical scale? Exploring this theme further through broader, multinational studies that encompass larger-scale countries would be particularly compelling. In addition, though participation with returned questionnaires was satisfactory, this might have influenced the results, particularly those observed at 3 and 5 years post-KT. Indeed, the number of collected questionnaires decreased notably after the 5-year mark. Reliance on self-reported questionnaires for the primary end point of work reintegration could also introduce inherent reporting biases. The definition of work reintegration primarily adopts quantitative measures, overlooking other pertinent socioeconomic factors such as sick leave and productivity. We were unable to study any correlation between reintegration and the work characteristics at the time of KT, such as working conditions (physical and psychosocial) and type of work. These 2 factors might impact return to work after KT. Many of the recorded covariables may be interdependent, particularly those related to social integration (such as education, baseline work capacity, and marital status) and kidney disease (including vascular or diabetic primary disease, cardiovascular comorbidities, and dialysis duration). Some of these parameters likely share long-term dependencies that existed years before the onset of kidney disease, whereas others develop later due to ineffective disease and treatment management. Consequently, the full model may include overadjustment bias that cannot be fully resolved. The study population focuses on adults who are compelled to compete for a job after the KT, and unfortunately excludes adolescent participants and people close to retirement. Moreover, crucial treatment-associated factors such as rejection episodes and hospitalization complications were not examined despite their likely substantial impact on work reintegration. Finally, our comparative analysis excludes patients with advanced kidney disease who are not yet KT recipients, limiting the comprehensiveness of our insights into work maintenance and reintegration within this patient cohort.

In conclusion, our findings showed favorable patient and graft outcomes, coupled with enhanced work reintegration at the 1-year mark post-KT for recipients of rural residency compared to those from suburban and urban settings. These results hold significant implications for transplantation decisions, particularly if the remote location of recipients might hinder their access to transplantation.

## Appendix

### List of the Swiss Transplant Cohort Study

The members of the Swiss Transplant Cohort Study are: Patrizia Amico, Adrian Bachofner, Vanessa Banz, Sonja Beckmann, Guido Beldi, Christoph Berger, Ekaterine Berishvili, Annalisa Berzigotti, Pierre-Yves Bochud, Sanda Branca, Heiner Bucher, Anne Cairoli, Emmanuelle Catana, Yves Chalandon, Sabina De Geest, Sophie De Seigneux, Michael Dickenmann, Joëlle Lynn Dreifuss, Michel Duchosal, Thomas Fehr, Sylvie Ferrari-Lacraz, Jaromil Frossard, Christian Garzoni, Déla Golshayan, Nicolas Goossens, Fadi Haidar, Jörg Halter, Dominik Heim, Christoph Hess, Sven Hillinger, Hans Hirsch, Patricia Hirt, Linard Hoessly, Günther Hofbauer, Uyen Huynh-Do, Franz Immer, Michael Koller, Andreas Kremer, Thorsten Krueger, Christian Kuhn, Bettina Laesser, Frédéric Lamoth, Roger Lehmann, Alexander Leichtle, Oriol Manuel, Hans-Peter Marti, Michele Martinelli, Valérie McLin, Katell Mellac, Aurélia Merçay, Karin Mettler, Nicolas Müller, Ulrike Müller-Arndt, Beat Müllhaupt, Mirjam Nägeli, Graziano Oldani, Manuel Pascual, Jakob Passweg, Rosemarie Pazeller, Klara Posfay-Barbe, David Reineke, Juliane Rick, Anne Rosselet, Simona Rossi, Rössler, Silvia Rothlin, Frank Ruschitzka, Thomas Schachtner, Stefan Schaub, Alexandra Scherrer, Dominik Schneidawind, Aurelia Schnyder, Macé Schuurmans, Simon Schwab, Thierry Sengstag, Federico Simonetta, Jürg Steiger, Guido Stirniman, Ueli Stürzinger, Christian Van Delden, Jean-Pierre Venetz, Jean Villard, Julien Vionnet, Madeleine Wick, Markus Wilhlem, and Patrick Yerly.

## Disclosure

All the authors declared no competing interests.

## Supplementary Material

Supplementary File (PDF) **Figure S1.** Causal diagram analysis of dependent and independent variables on outcomes ([A] 1-year work capacity, [B] composite of death and graft loss). **Figure S2.** Selection procedure for study participants. **Table S1.** Marital status stratified according to urbanization status (urban, suburban, rural). **Table S2.** Driving distance in kilometers and travel time in minutes to closest HD and KT center stratified according to urbanization status (urban, suburban, rural). **Table S3.** Work load at (A) 3 years and (B) 5 years after KT assessed from a reduced model (urbanization) and a full model (urbanization, age, gender, higher education, marital status, and work capacity at baseline). **Table S4.** Recipients and graft survival rates at 1-, 3-, 5-, and 10-years post-KT, stratified according to urbanization status (urban, suburban, rural). **Questionnaire of the Swiss Transplant Cohort Study (STCS).**

## Footnotes

Supplementary File (PDF)
**Figure S1.** Causal diagram analysis of dependent and independent variables on outcomes ([A] 1-year work capacity, [B] composite of death and graft loss). **Figure S2.** Selection procedure for study participants. Frequencies represent number of returned questionnaires at indicated time points (numerator) relative to the patients at risk (patients alive with functioning graft at the indicated time points, (denominator)). **Table S1.** Marital status stratified according to urbanization status (urban, suburban, rural). **Table S2.** Driving distance in kilometers and travel time in minutes to closest HD and KT center stratified according to urbanization status (urban, suburban, rural). **Table S3.** Work load at (A) 3 years and (B) 5 years after KT assessed from a reduced model (urbanization) and a full model (urbanization, age, sex, higher education, marital status, and work capacity at baseline). **Table S4.** Recipients and graft survival rates at 1-, 3-, 5-, and 10-years post-KT, stratified according to urbanization status (urban, suburban, rural).
**Questionnaire of the Swiss Transplant Cohort Study (STCS).**

## References

[1] Geography About Switzerland. https://www.eda.admin.ch/aboutswitzerland/en/home/umwelt/geografie.html.

[2] Boes S., Kaufmann C., Marti J. (2016). Sozioökonomische und kulturelle ungleichheiten im gesundheitsverhalten der Schweizer pevölkerung. https://www.obsan.admin.ch/de.

[3] Cartier T., Senn N., Cornuz J., Bourgueil Y., Kringos D.S., Boerma W.G.W., Hutchinson A. (2015). Building Primary Care in a Changing Europe: Case Studies [Internet].

[4] Bocchi F., Beldi G., Kuhn C., Storni F., Müller N., Sidler D. (2023). Impact of suboptimal donor to suboptimal recipient kidney transplant on delayed graft function and outcome. Front Transplant.

[5] Oniscu G.C., Ravanan R., Wu D. (2016). Access to transplantation and transplant outcome measures (ATTOM): study protocol of a UK wide, in-depth, prospective cohort analysis. BMJ Open.

[6] Petersen E., Baird B.C., Barenbaum L.L. (2008). The impact of employment status on recipient and renal allograft survival. Clin Transplant.

[7] Ambühl P.M. (2017). Aktuelle Erkenntnisse zur Schweizer Dialysepopulation. Hausarzt Praxis.

[8] Koller M.T., van Delden C., Müller N.J. (2013). Design and methodology of the Swiss Transplant Cohort Study (STCS): a comprehensive prospective nationwide long-term follow-up cohort. Eur J Epidemiol.

[9] Watters T.K., Glass B.D., Mallett A.J. (2023). Identifying the barriers to kidney transplantation for patients in rural and remote areas: a scoping review. J Nephrol.

[10] Goldberg D.S., French B., Forde K.A. (2014). Association of distance from a transplant center with access to waitlist placement, receipt of liver transplantation, and survival among US veterans. JAMA.

[11] Axelrod D.A., Dzebisashvili N., Schnitzler M.A. (2010). The interplay of socioeconomic status, distance to center, and interdonor service area travel on kidney transplant access and outcomes. Clin J Am Soc Nephrol CJASN.

[12] Fazekas C., Kniepeiss D., Arold N., Matzer F., Wagner-Skacel J., Schemmer P. (2021). Health-related quality of life, workability, and return to work of patients after liver transplantation. Langenbecks Arch Surg.

[13] Chou S., Deily M.E., Li S. (2014). Travel distance and health outcomes for scheduled surgery. Med Care.

[14] Kelly C., Hulme C., Farragher T., Clarke G. (2016). Are differences in travel time or distance to healthcare for adults in global north countries associated with an impact on health outcomes? A systematic review. BMJ Open.

[15] Urban Switzerland-cities and regions. Federal Statistical Office. https://www.bfs.admin.ch/bfs/en/home/statistics/cross-sectional-topics/city-statistics/urban-switzerland.html.

[16] WorldPop Population and SDG indicators by degree of urbanisation (DEBURBA). https://www.worldpop.org/current-projects/degurba/.

[17] Levey A.S., Stevens L.A., Schmid C.H. (2009). A new equation to estimate glomerular filtration rate. Ann Intern Med.

[18] Ferrario A., Verga F.C., Piolatto P.G., Pira E. (2014). Return to work after organ transplantation: a cross-sectional study on working ability evaluation and employment status. Transplant Proc.

[19] Eppenberger L., Hirt-Minkowski P., Dickenmann M. (2015). Back to work? Socioeconomic status after kidney transplantation. Swiss Med Wkly.

[20] Visser A., Alma M.A., Bakker S.J.L. (2022). Employment and ability to work after kidney transplantation in the Netherlands: the impact of preemptive versus non-preemptive kidney transplantation. Clin Transplant.

[21] Samaranayake C.B., Ruygrok P.N., Wasywich C.A., Coverdale H.A. (2013). Return to work after heart transplantation: the New Zealand experience. Transplant Proc.

[22] Henson J.B., Cabezas M., McElroy L.M., Muir A.J. (2023). Rates of employment after liver transplant: a nationwide cohort study. Hepatol Commun.

[23] Danuser B., Simcox A., Studer R., Koller M., Wild P., Psychosocial Interest Group, Swiss Transplant Cohort Study (2017). Employment 12 months after kidney transplantation: an in-depth bio-psycho-social analysis of the Swiss Transplant Cohort. PLoS One.

[24] Vieux L., Simcox A.A., Mediouni Z. (2019). Predictors of return to work 12 months after solid organ transplantation: results from the Swiss transplant cohort study. J Occup Rehabil.

[25] D’Egidio V., Mannocci A., Ciaccio D. (2019). Return to work after kidney transplant: a systematic review. Occup Med (Lond).

[26] Wlodarczyk E., Viklický O., Budde K. (2021). Analysis of factors affecting employment status of kidney transplant recipients in selected European Union Member States. Int J Environ Res Public Health.

