# Supplementary material for: Reintegration Into the Workforce After Kidney Transplantation Based on Urbanization Status in Switzerland
Source: Kidney Int Rep. 2024 Nov 6;10(2):565–73. doi: 10.1016/j.ekir.2024.10.029 (PMC11843122; doi:10.1016/j.ekir.2024.10.029)
Supplement: Supplementary File (PDF) — Figure S1. Causal diagram analysis of dependent and independent variables on outcomes ([A] 1-year work capacity, [B] composite of death and graft loss). Figure S2. Selection procedure for study participants. Table S1. Marital status stratified according to urbanization status (urban, suburban, rural). Table S2. Driving distance in kilometers and travel time in minutes to closest HD and KT center stratified according to urbanization status (urban, suburban, rural). Table S3. Work load at (A) 3 years and (B) 5 years after KT assessed from a reduced model (urbanization) and a full model (urbanization, age, gender, higher education, marital status, and work capacity at baseline). Table S4. Recipients and graft survival rates at 1-, 3-, 5-, and 10-years post-KT, stratified according to urbanization status (urban, suburban, rural). Questionnaire of the Swiss Transplant Cohort Study (STCS). [file mmc1.pdf]

Supplementary materials

Suppl. Table 1) Marital status stratified according to urbanization status (urban, suburban, rural).

	**Urbanization**				
	**Overall N = 1926**	**Urban N = 630 (33%)**	**Suburban N = 871 (45%)**	**Rural N = 425 (22%)**	**p-value <0.01**
Single	431 (25%)	163 (29%)	190 (24%)	78 (21%)	
Married	1,037 (61%)	302 (55%)	485 (63%)	250 (68%)	
Divorced	200 (12%)	82 (15%)	84 (11%)	34 (9.3%)	
Widow	27 (1.6%)	6 (1.1%)	17 (2.2%)	4 (1.1%)	

Suppl. Table 2) Driving distance in kilometers (km) and travel time in minutes (min) to closest HD and KT center stratified according to urbanization status (urban, suburban, rural). HD: Hemodialysis. KT: Kidney Transplantation. Km: Kilometers. Min: Minutes.

	**Urbanization**				
	**Overall N = 1926**	**Urban N = 630 (33%)**	**Suburban N = 871 (45%)**	**Rural N = 425 (22%)**	**p-value**
**HD center**					
Distance (km)	5.7 (2.8, 10.9)	2.9 (1.7, 4.5)	6.4 (3.7, 9.8)	12.1 (7.6, 17.9)	**< 0.001**
Travel time (min)	10.7 (6.8, 15.1)	7.6 (5.2, 10.6)	11.3 (7.7, 14.8)	15.1 (11.1, 21.2)	**< 0.001**
**KT center**					
Distance (km)	32.5 (11.6, 52.1)	29.0 (4.1, 51.2)	25.1 (11.6, 42.3)	46.2 (35.5, 67.2)	**< 0.001**
Travel time (min)	31.6 (19.1, 47.0)	28.5 (12.5, 45.2)	27.7 (18.9, 40.1)	42.6 (33.4, 55.8)	**< 0.001**

Suppl. Table 3) Work load at 3 years (a) and 5 years (b) after KT assessed from a reduced model (urbanization) and a full model (urbanization, age, gender, higher education, marital status, and work capacity at baseline). KT: Kidney Transplantation. LDT: Living Donor Transplantation. BL: Baseline. OR: Odds Ratio. CI: Confidence Interval.

a)

**3 YEARS**	**Minimal model**			**Full model**		
	OR	95% CI	p-value	OR	95% CI	p-value
**Urbanization**						
Urban	-	-		-	-	
Suburban	1.24	0.97, 1.59	0.08	1.22	0.97, 1.53	0.091
Rural	1.53	1.15, 2.05	**0.004**	1.28	0.98, 1.67	0.069
**Recipient age** (per decade)				0.72	0.65, 0.79	<0.001
**Recipient gender** (male)				1.63	1.33, 2.00	**<0.001**
**Higher education** (yes)				1.55	1.22, 1.99	**<0.001**
**Marital status** (married)				1.16	0.94, 1.44	0.2
**Work capacity at BL** (> 50%)				4.42	3.60, 5.44	**<0.001**

b)

**5 YEARS**	**Minimal model**			**Full model**		
	OR	95% CI	p-value	OR	95% CI	p-value
**Urbanization**						
Urban	-	-		-	-	
Suburban	1.10	0.82, 1.46	0.5	1.09	0.83, 1.41	0.5
Rural	1.38	0.97, 1.95	0.073	1.24	0.90, 1.69	0.2
**Recipient age** (per decade)				0.66	0.59, 0.74	**<0.001**
**Recipient gender** (male)				1.51	1.19, 1.92	**<0.001**
**Higher education** (yes)				1.42	1.06, 1.91	**0.019**
**Marital status** (married)				1.07	0.83, 1.39	0.6
**Work capacity at BL** (> 50%)				3.62	2.84, 4.62	**<0.001**

Suppl. Table 4) Recipients and graft survival rates at 1, 3, 5 and 10 years post-KT stratified according to urbanization status (urban, suburban, rural).

	**1 Years**	**3 Years**	**5 Years**	**10 Years**
**Urban**	97% (96%-98%)	92% (90%-94%)	89% (86%-92%)	77% (72%-83%)
**Suburban**	95% (94%-97%)	93% (91%-95%)	88% (86%-91%)	76% (71%-81%)
**Rural**	95% (93%-97%)	92% (90%-95%)	88% (84%-92%)	76% (69%-82%)

Fig suppl. 1.

a)

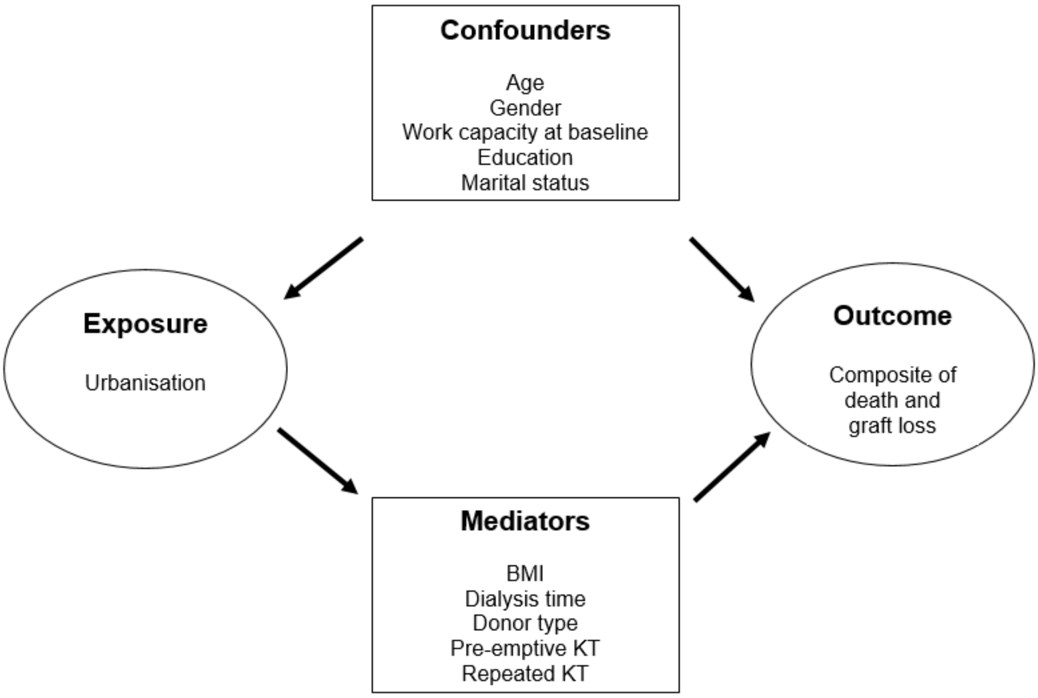

b)

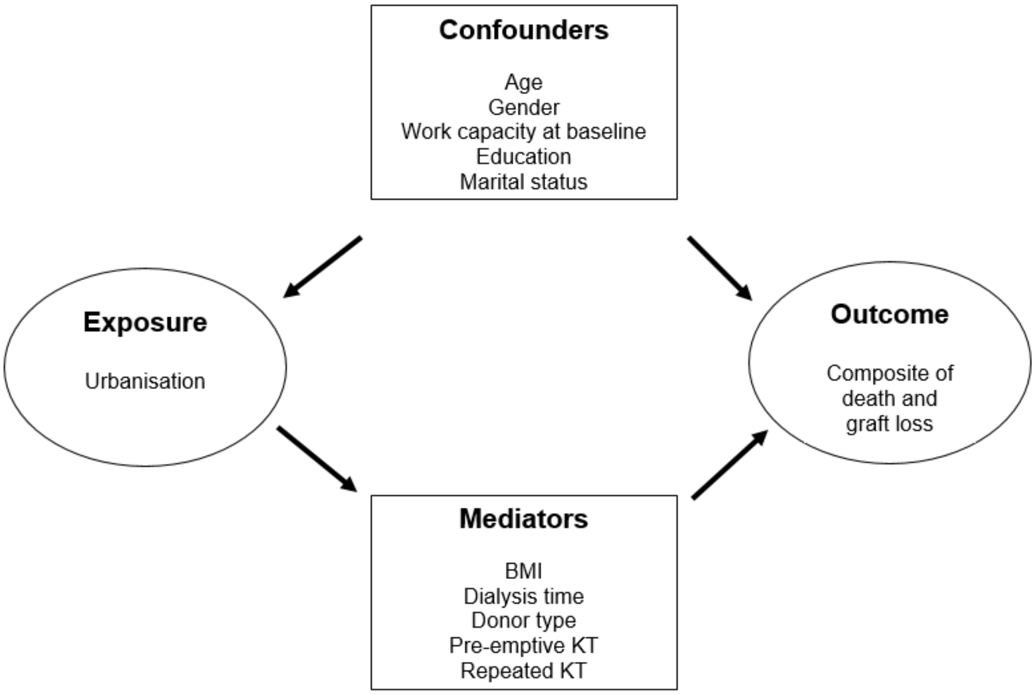

Causal diagram analysis of dependent and independent variables on outcomes (1-year work capacity (a), composite of death and graft loss (b)). Variables are grouped in mediators and cofounders based on expert knowledge. KT: Kidney Transplantation. BMI: Body Mass Index.

Fig suppl. 2.

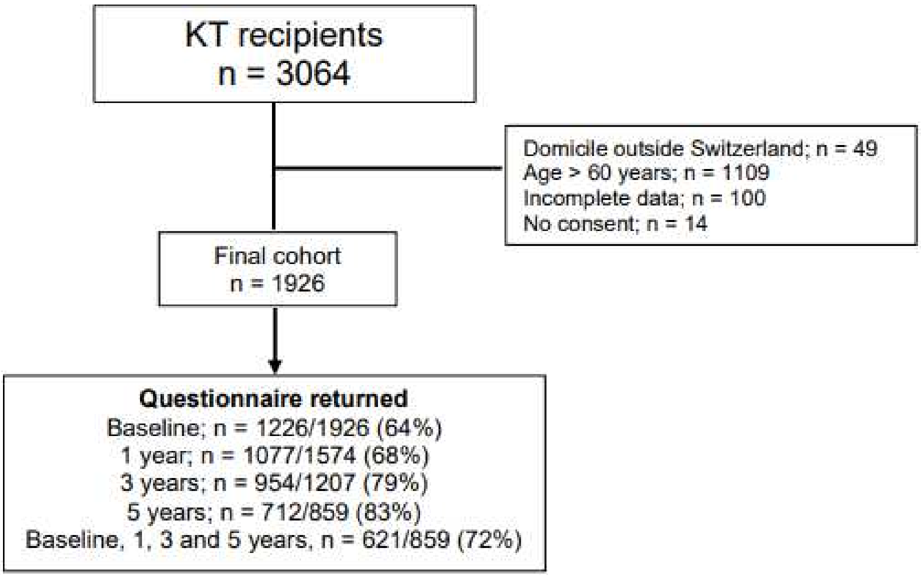

Selection procedure for study participants. Frequencies represent number of returned questionnaires at indicated time points (numerator) relative to the patients at risk (patients alive with functioning graft at the indicated time points, (denominator)). KT: Kidney Transplantation.

Questionnaire

of the Swiss Transplant Cohort Study (STCS)

Pre-transplantation

Instruction for completing the questionnaire

1. Please choose the answer that best fits your current situation (please choose only **one** answer)
2. Please mark your answer with a cross ☒ in the box
3. Please do not place a cross outside of the provided answer boxes

Please bring or send the completed questionnaire in the pre-stamped and pre-addressed envelope within the next 10 days.

Thank you!

Please leave this box empty, the center will fill it in.

Date received:
SOAS – Nr.:
STCS – Nr.:
Notes:

SWISS TRANSPLANT COHORT STUDY

STCS

0. Completion date

Please fill in the completion date of the questionnaire.

☐☐/☐☐/☐☐☐☐ (dd/mm/yyyy)

1. Educational level

Please specify your **highest** completed educational degree.
(please select only one answer)

☐1 No completed school or professional education (less than 9 formation years)
☐2 Mandatory school (primary/secondary/junior high/district school) (9 formation years)
☐3 Apprenticeship or full-time vocational school (10-13 formation years)
☐4 Diploma qualifying for university admission (Matura) (13 formation years)
☐5 Higher professional education (e.g. master craftsman diploma, federal diploma) (14-16 formation years)
☐6 Higher technical or commercial school (e.g. school for social work, school for engineering) (14-18 formation years)
☐7 University degree (e.g. bachelor or master of science) (16 or more formation years)
☐8 Other: ________________________________
☐9 I do not want to answer

2. Professional status

Please specify your **current primary** occupation. *(please select only one answer)*

☐1 Self-employed (e.g. own store, own company, freelance)
☐2 Working in a relative's firm or business
☐3 Apprentice, trainee (e.g. with training contract)
☐4 Director, manager, procurator
☐5 Middle/lower management (e.g. office manager, branch manager)
☐6 Employee in another function (e.g. staff, blue-collar worker)
☐7 Houseman/-wife in your own home
☐8 Student, scholar
☐9 Retirement pensioner
☐10 Invalidity pensioner (AI/IV)
☐11 Other: ________________________________
☐12 I do not want to answer

3. Working capacity

Please specify your **average** working/earning capacity during the past **6 months.** 100% equals full-time workload. *(please select only one answer)*

☐1 More than 80% workload
☐2 Between 51% and 80% workload
☐3 Between 21% and 50% workload
☐4 Between 1% and 20% workload
☐5 0% workload
☐6 I do not want to answer

If **0%** working capacity, please indicate the **main** reason *(please select only one answer)*

☐1 Houseman/-wife in your own home
☐2 In education
☐3 Retirement pensioner
☐4 Illness
☐5 Unemployed
☐6 Invalidity pensioner (AI/IV)
☐7 Other reason:________________
☐8 I do not want to answer

4. Relationship status

What is your **current** status?
(please select the most appropriate answer for your current situation)

☐1 Single
☐2 Married/stable partnership/cohabitation
☐3 Widow/widower
☐4 Divorced/dissolved partnership/separated
☐5 I do not want to answer

5. Socio-economic status

What is the **current** available monthly budget (after tax deductions) of your household? *(please select only one answer)*

☐1 Less than Fr. 4500.-
☐2 Between Fr. 4500.- and 6000.-
☐3 Between Fr. 6001.- and 9000.-
☐4 Over Fr. 9000.-
☐5 I do not want to answer

SWISS TRANSPLANT COHORT STUDY

STCS

6. Sleep quality[1]

On a scale from 0 to 10, how would you rate your sleep quality overall in the **past 4 weeks**? *(please select only one answer)*

Very poor										Very good
0	1	2	3	4	5	6	7	8	9	10
☐	☐	☐	☐	☐	☐	☐	☐	☐	☐	☐

7. Daytime sleepiness

On a scale from 0 to 10, how would you rate your daytime sleepiness overall in the **past 4 weeks**? *(please select only one answer)*

Not at all sleepy										Very sleepy
0	1	2	3	4	5	6	7	8	9	10
☐	☐	☐	☐	☐	☐	☐	☐	☐	☐	☐

8. Medication adherence[2]

Taking medication regularly can be challenging. Therefore, we would like to ask you the following questions about your medication taking behavior:

a. How often did you **miss** a dose of your medication in the **past 4 weeks**? *(please select only one answer)*

- ☐1 This question does not apply to me because I do not take regular prescribed medication *(=>please go on to question no.9)*
- ☐2 Never (I always took my medication) *(=>please go on to question no.9)*
- ☐3 Once a month
- ☐4 Once every 2 weeks
- ☐5 Once a week
- ☐6 More than once a week
- ☐7 Every day (I missed my medication every day)

b. Did you **miss** more than one consecutive dose of your medication in the **past 4 weeks**? *(please select only one answer)*

- ☐1 Yes
- ☐2 No

---

[1] Elder, S. J.et al., Nephrology, Dialysis, Transplantation,2008; 23(3), 998-1004.

[2] © Basel Assessment of Adherence with Immunosuppressive Medication (BAASIS)

SWISS TRANSPLANT COHORT STUDY

9. Smoking[3]

Do you smoke? *(please select only one answer)*

- ☐1 Yes
- ☐2 No, I stopped **less** than one year ago
- ☐3 No, I stopped **more** than one year ago
- ☐4 No, I never smoked
- ☐5 I do not want to answer

10. Hard drug use

Do you consume hard drugs (e.g. Heroin, Cocaine)?
(please select only one answer)

- ☐1 Yes
- ☐2 No, I stopped **less** than one year ago
- ☐3 No, I stopped **more** than one year ago
- ☐4 No, I never consumed hard drugs
- ☐5 I do not want to answer

11. Physical exercise[4]

Do you participate in regular physical activity (e.g. walking, cycling, cleaning the house, yard work)? *(please select only one answer)*

- ☐1 No *(=>please go on to question no.12)*
- ☐2 Yes

If yes

a. How many days per week? ___days

b. How many minutes per session on average? ___minutes

c. How hard do you exert yourself during your physical activity? *(please select only one answer)*

- ☐1 Very easy
- ☐2 Easy
- ☐3 Somewhat hard
- ☐4 Hard
- ☐5 Very Hard

[3] Elzi, L. et al., Antivir Ther, 2006; 11(6), 787-795.
[4] Nielens, H.et al., Nephrol.Dial.Transplant.,2001; 16(1), 134-140.

12. Sun protection[5]

1. Occupational sun exposure. In the summer, on average, how many hours are you outside per day between 10 am and 4 pm on **weekdays** (Monday-Friday)? *(please select only one answer)*

   ☐1 30 minutes or less
   ☐2 31 minutes to 1 hour
   ☐3 2 hours
   ☐4 3 hours
   ☐5 4 hours
   ☐6 5 hours
   ☐7 6 hours

2. Sun exposure during leisure time. In the summer, on average, how many hours are you outside per day between 10 am and 4 pm on **weekend days** (Saturday & Sunday)? *(please select only one answer)*

   ☐1 30 minutes or less
   ☐2 31 minutes to 1 hour
   ☐3 2 hours
   ☐4 3 hours
   ☐5 4 hours
   ☐6 5 hours
   ☐7 6 hours

3. Sun protection behavior. How often do you wear **sunscreen**? *(please select only one answer)*

   ☐1 Never
   ☐2 Rarely
   ☐3 Sometimes
   ☐4 Often
   ☐5 Always

4. Sun protection behavior. How often do you wear a **hat**? *(please select only one answer)*

   ☐1 Never
   ☐2 Rarely
   ☐3 Sometimes
   ☐4 Often
   ☐5 Always

[5] Glanz, K. et al. Archives of Dermatology, 2008;144(2):217-222.

13. Trust in the transplant team[6]

How much do you trust your transplant team, where ’0’ is ’not at all’ and ’10’ is ’completely’? *(please select only one answer)*

Not at all										Completely
0	1	2	3	4	5	6	7	8	9	10
☐	☐	☐	☐	☐	☐	☐	☐	☐	☐	☐

14. Perceived health status[7]

By placing a cross in one box in each group below, please indicate which statements best describe your own health state **today**.

1. Mobility

☐1 I have no problems in walking about
☐2 I have some problems in walking about
☐3 I am confined to bed

2. Self-care

☐1 I have no problems with self-care
☐2 I have some problems washing or dressing myself
☐3 I am unable to wash or dress myself

3. Usual activities (e.g. working, study, household chore, family or leisure activities)

☐1 I have no problems with performing my usual activities
☐2 I have some problems with performing my usual activities
☐3 I am unable to perform my usual activities

4. Pain/Discomfort

☐1 I have no pain or discomfort
☐2 I have moderate pain or discomfort
☐3 I have extreme pain or discomfort

5. Anxiety/Depression

☐1 I am not anxious or depressed
☐2 I am moderately anxious or depressed
☐3 I am extremely anxious or depressed

---

[6] Saha, S. et al. Aids Patient Care and STDS, 2010;24(7): 415-420.

[7] Standard questionnaire EQ-5D™ © 1990 EuroQol Group.

SWISS TRANSPLANT COHORT STUDY

STCS

To help people say how good or bad a health state is, we have drawn a scale (rather like a thermometer) on which the best state you can imagine is marked 100 and the worst state you can imagine is marked 0.

We would like you to indicate on this scale how good or bad your own health is **today**, in your opinion. Please do this by drawing a line from the box below to whichever point on the scale indicates how good or bad your health state is **today**.

Best imaginable health state

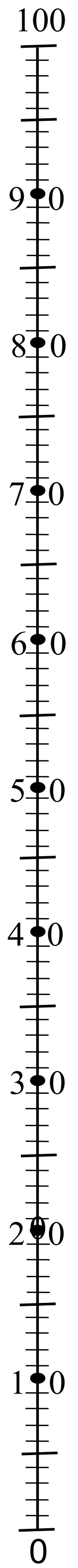

Worst imaginable health state

Current health state

Example:

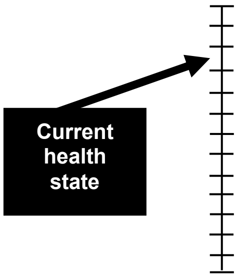

15. Quality of life[8]

Could you please mark the line below at the place which best reflects your situation of the **past week**. The more your situation was close to perfect quality of life, the further you place the mark to the right. The more your situation was close to worst imaginable quality of life, the further you place the mark to the left.

Worst imaginable quality of life — Perfect quality of life

16. Depressive symptomatology[9]

Please read each item below and cross the box next to the reply that comes closest to how you have been feeling in the **past week.** Don't take too long over your replies; your immediate reaction to each item will probably be more accurate than a long, thought-out response.

1. I still enjoy the things I used to enjoy
   - ☐1 Definitely as much
   - ☐2 Not quite so much
   - ☐3 Only a little
   - ☐4 Not at all

2. I can laugh and see the funny side of things
   - ☐1 As much as I always could
   - ☐2 Not quite so much now
   - ☐3 Definitely not so much now
   - ☐4 Not at all

3. I feel cheerful
   - ☐1 Not at all
   - ☐2 Not often
   - ☐3 Sometimes
   - ☐4 Most of the time

4. I feel as if I am slowed down
   - ☐1 Nearly all of the time
   - ☐2 Very often
   - ☐3 Sometimes
   - ☐4 Not at all

[8] De Boer A.G. et al., Quality of Life Research, 2004; 13(2), 311-320.

[9] Zigmond, A.S., Snaith, R.P., Acta Psychiatrica Scandinavica, 1983; 67(6): 361-370.

5. I have lost interest in my appearance
   - ☐1 Definitely
   - ☐2 I don't take as much care as I should
   - ☐3 I may not take quite as much care
   - ☐4 I take just as much care as ever

6. I look forward with enjoyment to things
   - ☐1 As much as I ever did
   - ☐2 Rather less than I used to
   - ☐3 Definitely less than I used to
   - ☐4 Hardly at all

7. I can enjoy a good book or radio or TV program
   - ☐1 Often
   - ☐2 Sometimes
   - ☐3 Not often
   - ☐4 Very seldom

17. Stress[10]

Stress means a situation in which a person feels tense, restless, nervous or anxious or is unable to sleep at night because his/her mind is troubled all the time: Do you feel this kind of stress **these days**?

On a scale from 1 to 5, how would you rate this kind of stress **these days**? *(please select only one answer)*

Not at all stressed	Only a little Stressed	To some extent stressed	Rather much stressed	Very much stressed
1	2	3	4	5
☐	☐	☐	☐	☐

18. Filling in the questionnaire

Please specify if you filled in the questionnaire by yourself.
(please select only one answer)

☐1 I filled in the questionnaire by myself
☐2 I received help filling in the questionnaire

[10] Elo, A.L. et al. al, Scand J Work Environ Health 2003; 29: 444-51.
